# The illusory correlation between geometrical similarity and physician satisfaction in heart auto‐segmentation revealed by a physician‐blind test

**DOI:** 10.1002/acm2.70642

**Published:** 2026-06-15

**Authors:** Jae Choon Lee, Eun Jeong Heo, Song Heui Cho, Dong Yun Lee, Kyung Hwan Chang, Jang Bo Shim, Nam Kwon Lee, Suk Lee

**Affiliations:** ^1^ Department of Radiation Oncology, College of Medicine Korea University Seoul Republic of Korea; ^2^ Department of Medical Physics Kyonggi University Gyeonggi Republic of Korea; ^3^ Department of Medical Physics Graduate School of Korea University Sejong‐si Chungcheongnam‐do Republic of Korea; ^4^ Department of Sales and CS OncoSoft Seoul Republic of Korea; ^5^ Department of Radiologic Science Far East University Eumseong‐gun Chungcheongbuk‐do Republic of Korea; ^6^ Department of Radiation Oncology Guro Hospital Korea University Medical Center Seoul Republic of Korea

**Keywords:** adaptive radiation therapy (ART), auto‐segmentation, geometric similarity index, physician satisfaction, physician‐blind test

## Abstract

**Background:**

Auto‐segmentation tools are essential in adaptive radiation therapy (ART). While evaluation typically relies on geometric metrics like Dice similarity coefficient (DSC), high scores do not always translate to clinical acceptability.

**Purpose:**

This study investigated the “illusory correlation” between geometric indices and physician satisfaction and aimed to identify critical anatomical substructures that dictate clinical judgment.

**Methods:**

Heart auto‐segmentation was performed for 30 left‐sided breast cancer patients using pre‐built and on‐site trained fully convolutional dense network (FCDN) models. Geometric similarity was assessed via DSC, mean surface distance (MSD), and 95th percentile Hausdorff Distance (HD95). Clinical satisfaction was quantified through a physician‐blind test involving 17 anatomical items. Pearson correlation coefficients (PCC) and conditional probability (*P(B|A)*) were used to analyze the relationship between substructure accuracy and overall clinical acceptance.

**Results:**

Both models showed high geometric similarity (mean DSC ∼ 0.95), but clinical satisfaction differed drastically: the pre‐built model had a 3.3% acceptance rate, while the on‐site model achieved 93.3%. PCC analysis failed to show significant correlations between geometric metrics and satisfaction after multiple testing corrections. However, conditional probability analysis revealed that the cranial/caudal borders and the superior vena cava (SVC) were the primary determinants of satisfaction, with *P(B|A)* values up to 0.95. Conversely, while coronary arteries showed the greatest geometric improvement in the on‐site model, their individual success did not guarantee overall clinical acceptance (*P(B|A)* ∼ 0.42–0.52), explaining the disconnect between standard metrics and clinical judgment.

**Conclusions:**

Geometric metrics alone are insufficient to validate auto‐segmentation for clinical use. Clinical acceptance is driven by specific critical substructures, particularly boundary regions, rather than global volumetric similarity. We propose a “two‐guideline strategy” that prioritizes these high‐impact regions and establishes quantitative gateways for model commissioning, providing a more robust framework for quality assurance (QA) in ART.

## 1 INTRODUCTION

Recently, a growing number of commercial auto‐segmentation tools have emerged for use in radiation oncology, forming a crucial component of adaptive radiation therapy (ART). The success of ART inherently relies on the accurate performance of both auto‐segmentation and auto‐planning. Auto‐segmentation, as the initial step, demands precise organ delineation,^1^, ^2^, ^3^, ^4^ while auto‐planning, the subsequent step, necessitates accurate prediction of safe dose distribution.^5^, ^6^, ^7^, ^8^, ^9^ Given that accurate organ delineation significantly impacts treatment outcomes,^10^ the development of these auto‐segmentation tools has primarily focused on enhancing their quality to match the contouring standards established by radiation oncologists (ROs).

To evaluate auto‐segmentation outcomes, developers commonly utilize various geometrical similarity metrics. The Dice Similarity Coefficient (DSC) is computed based on the overlapping area between two contours.^11^ The MSD quantifies the average distance between two contours,^12^ while the 95th percentile Hausdorff Distance (HD95) represents the 95th percentile of distances between them.^13^ However, an ongoing question persists regarding whether favorable scores in these geometrical similarity indices directly correlate with ROs’ satisfaction with auto‐segmentation results. To investigate this, physician‐blind tests are frequently conducted to assess ROs' satisfaction with auto‐segmentation outcomes.

For example, Chung et al.^14^ reported a DSC value of 0.95 for heart segmentation, accompanied by a physician satisfaction score of 9/10 in a physician‐blind test. Conversely, Baroudi et al.^7^ observed that for duodenum auto‐segmentation, despite a DSC of 0.61, physician satisfaction was 4/5. These findings collectively suggest that high geometrical similarity metrics may not always directly correlate with ROs’ satisfaction. Nevertheless, research exploring the direct relationship between these two critical aspects remains limited.

Specifically, Mao et al.^15^ conducted a study comparing auto‐segmentation with reference contours for patients with lung cancer undergoing online ART. In their methodology, daily cone‐beam computed tomography (CBCT) images, acquired prior to treatment, were scanned to generate auto‐segmentations of organs at risk (OARs). Subsequently, using these auto‐segmentation results and the reference contours, the accumulated dose to the OARs was calculated upon completion of all treatments. Their study reported maximum daily dose differences to the heart, specifically a mean heart dose (MHD) of 4%, V50% of 32%, V30Gy of 27%, and D0.03cc of 1%. For the accumulated dose, the differences observed were an MHD of 2%, V50% of 3%, V30Gy of 5%, and D0.03cc of 4%. These findings underscore that auto‐segmentation results ultimately play a crucial role in the outcome of radiation therapy. Consequently, the clinical accuracy of these contours must be determined by the expert judgment of ROs.

Therefore, this study aimed to analyze the correlation between geometrical similarity indices and physician‐blind test results concerning physician satisfaction in heart auto‐segmentation. To investigate this relationship, we specifically developed a physician‐blind test for heart auto‐segmentation and analyzed its outcomes.

## 2 METHODS

**FIGURE 1:**
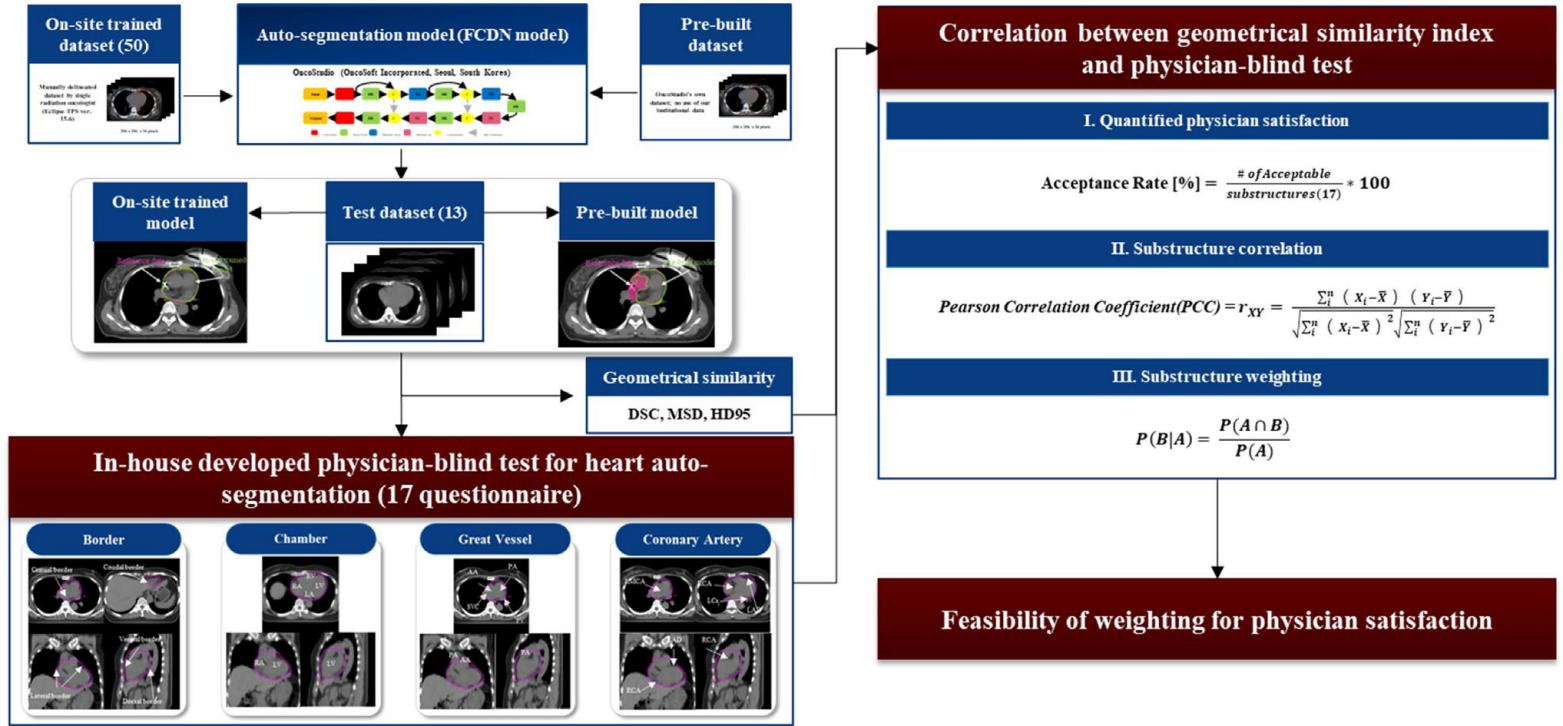
Workflow of this study. This figure illustrates the workflow designed to analyze the correlation between physician satisfaction and geometrical similarity in heart auto‐segmentation. Two distinct auto‐segmentation models were utilized. An in‐house physician‐blind test was developed to quantitatively assess physician satisfaction. Subsequently, three methods were employed to analyze the correlation between physician satisfaction and geometrical similarity.

As illustrated in Figure 1, our study workflow was designed to analyze the correlation between physician satisfaction and geometrical similarity indices in heart auto‐segmentation. To achieve this objective, we performed auto‐segmentation using two distinct models. Concurrently, we developed an in‐house physician‐blind test to quantitatively assess physician satisfaction. Subsequently, from the auto‐segmentation results, we calculated various geometrical similarity indices and conducted an in‐depth analysis of their correlations with quantified physician satisfaction.

### 2.1 Auto‐segmentation

Auto‐segmentation was performed on 30 patients with left breast cancer using two distinct models. The first was a pre‐built model, representing a standard model typically integrated into commercial auto‐segmentation tools. The on‐site training dataset consisted of planning computed tomography (CT) scans from 50 randomly selected patients with left‐sided breast cancer treated at our institution between 2023 and 2024. Inclusion criteria were adjuvant radiotherapy after breast‐conserving surgery and availability of complete RTSTRUCT data. All images were anonymized prior to analysis, and this retrospective study was approved by the institutional review board (IRB No. 2025AN0547), with informed consent waived.

The heart auto‐segmentation in this study was performed using the OncoStudio software platform (OncoSoft, Seoul, Korea). Both models were based on the Fully Convolutional Dense Network (FCDN) architecture^16^ implemented within the OncoStudio environment. The network was designed to perform segmentation in a 2‐dimensional slice‐based manner, processing axial CT slices to generate heart contours. The FCDN incorporates dense blocks that iteratively concatenate features to optimize parameter efficiency and promote feature reuse. Both downsampling and upsampling paths consist of multiple transitions; each downsampling transition applies a 1 × 1 convolution followed by 2 × 2 max pooling, whereas each upsampling transition uses 3 × 3 transposed convolutions. Additionally, direct skip connections between corresponding layers in the downsampling and upsampling paths enhance spatial detail recovery and improve fine‐grained segmentation accuracy.

During preprocessing, all CT images were resampled to achieve a uniform voxel spacing of 1.0 × 1.0 × 3.0 mm^3^, and the image intensity values were truncated to a range of −140 to 260 Hounsfield units, which were then linearly normalized between 0 and 1. For network training, two‐dimensional axial slices with a resolution of 256 × 256 pixels were used, with a batch size of 10. The FCDN was trained using a combined loss function comprising the sum of cross‐entropy and dice loss. Optimization was performed using the Adam optimizer with a learning rate of 0.0002, and the training process was conducted for 100 epochs on the CUDA 11.8 platform using an NVIDIA RTX A5000 24GB GPU. The auto‐segmentation results from both models were then evaluated against retrospective contour data by calculating the geometrical similarity indices (DSC, MSD, HD95).

### 2.2 Physician‐blind test

The physician‐blind test was conducted by two RO including our (RO1) and another (RO2) institution and a medical physicist (MP), both possessing significant experience in radiation oncology. The test was administered to the same 30 patients with left breast cancer on whom auto‐segmentation had been performed.

#### 2.2.1 Physician‐blind test design

To quantitatively assess physician satisfaction, we developed a novel approach that deviates from conventional physician‐blind tests. Traditional methods typically only evaluate overall satisfaction with an auto‐segmentation result. However, this approach can be challenging to quantify effectively because it frequently fails to account for the specific anatomical features of OARs and, instead, yields a general, subjective measure.

Our study addresses this limitation by segmenting and individually evaluating specific anatomical regions of the heart. The physician‐blind test for heart auto‐segmentation in this study comprised 17 detailed items, along with an additional item for overall satisfaction. This comprehensive design allows for a direct comparison between our quantitative method and the conventional approach. For each item, reviewers were asked to respond with either “Acceptable (one point)” or “Unacceptable (zero points).” The “Acceptable” or “Unacceptable” judgment for each substructure and the final overall satisfaction was based on whether the automated contour could be used immediately for treatment planning without any manual editing, as defined by the institutional standard contouring guidelines. Before the blind test, an observer calibration and training process was conducted between the RO and MP. This included joint review and discussion of several training cases to harmonize the definition of a clinically acceptable heart contour and its substructures according to institutional protocol, ensuring consistency in judgment. The 17 items were further categorized into four main groups:

- Borders: This category evaluates the cranial border, caudal border, ventral border, dorsal border, and lateral border.
- Chambers: This includes assessment of the left atrium (LA), left ventricle (LV), right atrium (RA), and right ventricle (RV).
- Great Vessels: This category focuses on the ascending aorta (AA), pulmonary artery (PA), pulmonary vein (PV), and superior vena cava (SVC).
- Coronary Arteries: This covers the left main coronary artery (LCA), left anterior descending coronary artery (LAD), left circumflex coronary artery (LCx), and right coronary artery (RCA).

This comprehensive assessment framework was designed based on the Radiation Therapy Oncology Group guidelines^17^, ^18^, ^19^ and further informed by our institution's clinical experience. These substructures were selected based on established RTOG heart contouring guidelines and their reported relevance to cardiac dose–response relationships in clinical toxicity literature.

#### 2.2.2 Quantifying physician‐blind test

To quantitatively analyze the results of the physician‐blind test, we calculated the acceptance rate. This rate represents the percentage of satisfactory responses obtained across the 17 individual items of the physician‐blind test. The formula for calculating the acceptance rate is as follows:

(1) $$\begin{array}{cc} & Acceptance\;rate\;\left(\%\right)\\ & \;=\frac{Number\;of\;acceptable\;substructure}{Total\;substructures\left(17\right)}\times 100 \end{array}$$

### 2.3 Correlation between geometrical similarity and physician‐blind test

In this study, three analytical methods were employed to ascertain the correlation between the geometrical similarity indices and the physician‐blind test. First, we sought to determine the relationship between the geometrical similarity indices and overall physician satisfaction. Second, we aimed to identify which substructures correlate with the geometrical similarity index. Third, we investigated how each substructure's acceptability relates to overall physician satisfaction.

#### 2.3.1 Quantified physician satisfaction

To investigate the relationship between geometrical similarity indices and physician satisfaction, we categorized the physician‐blind test results into two distinct groups based on the overall satisfaction response. The “Acceptable” Group comprised cases where the overall satisfaction with the auto‐segmentation result was marked as “Acceptable,” whereas the ‘Unacceptable’ Group included cases where the overall satisfaction was marked as ‘Unacceptable.’ For both groups, we then compared their respective acceptance rates and the calculated geometrical similarity indices.

#### 2.3.2 Substructure correlation

To determine which substructures correlate with geometrical similarity, we analyzed the Pearson correlation coefficient (PCC)^20^ between the acceptance rate for each of the 17 individual substructures and the corresponding geometrical similarity indices.

#### 2.3.3 Substructure weighting

To analyze the relationship between the acceptability of individual substructures and overall physician satisfaction, we employed conditional probability.

(2) $$P\left(B|A\right)=\frac{P\left(A\cap B\right)}{P\left(A\right)}$$

Here, *P(A)* represents the acceptability probability of each substructure (i.e., the probability that a specific substructure is deemed “acceptable”), while *P(B)* represents the acceptability probability of overall satisfaction (i.e., the probability that the entire auto‐segmentation result is deemed “acceptable” by the physician). By analyzing the conditional probability, specifically *P(B∣A)*, we can ascertain the likelihood of overall satisfaction being acceptable given that a particular substructure is acceptable. This approach helps us identify which specific substructures, when contoured acceptably, are critical determinants of the RO's overall satisfaction with the auto‐segmentation. In essence, it reveals the substructures whose accurate delineation most significantly impacts overall physician approval.

### 2.4 Statistical analysis

Statistical analyses were performed using R (version 4.2.2; R Foundation for Statistical Computing, Vienna, Austria). Two‐sample t‐tests (or Welch's t‐test when variances were unequal) and Mann–Whitney U tests were applied as appropriate. Fisher's exact test was used to compare inter‐observer differences. PCC were calculated for exploratory analysis, and multiple testing correction was applied using the Benjamini–Hochberg (BH) method (FDR *q* < 0.05).

#### 2.4.1 Comparison of geometrical metrics

The geometrical metrics (DSC, MSD, HD95) of the heart between the “Acceptable” group and the ‘Unacceptable’ group, as determined by the overall physician satisfaction, were compared. Based on the observed heterogeneity of variance in the data, a two‐sample *t*‐test assuming equal or unequal variances was primarily used. The Mann‐Whitney *U*‐test was additionally employed for nonparametric comparison to validate the results. A *p*‐value < 0.05 was considered statistically significant.

#### 2.4.2 Comparison of acceptance rates

The difference in the overall acceptance rate between the RO and the MP for the pre‐built model was analyzed using the Fisher's Exact Test to assess inter‐rater reliability on the clinical usability threshold.

#### 2.4.3 Correlation analysis

The PCC analysis for the 17 substructures was performed solely for exploration and hypothesis‐generating purposes. but, to mitigate the risk of false positive correlations in this exploratory PCC analysis, multiple comparison correction was performed using the BH adjustment.

#### 2.4.4 Conditional probability analysis

The significance of each substructure is reflected through conditional probability analysis Substructures with high conditional probabilities indicate strong critical effects on overall satisfaction. Additionally, the relationship with overall satisfaction was further validated by calculating expected values.

## 3 RESULTS

### 3.1 Auto‐segmentation

Heart auto‐segmentation was performed on 30 patients with left breast cancer using two different models. Both models yielded an identical average DSC of 0.948 ± 0.02 at pre‐built model and 0.954 ± 0.01 at on‐site model (*p*‐value: 0.13). For the average MSD, a difference of 0.23 mm was observed between the two models (pre‐built model: 1.606 ± 0.583, on‐site model: 1.373 ± 0.561, p‐value: 0.12). Similarly, the average HD95 showed a difference of 1.28 mm (pre‐built model: 6.334 ± 2.820, on‐site model: 5.054 ± 1.747, p‐value: 0.04). However, it is important to note that the differences in these geometrical similarity indices between the two models were not statistically significant except HD95.

### 3.2 Physician‐blind test

The physician‐blind test was conducted on 30 patients with left breast cancer by both two RO and an MP. The RO1 expressed dissatisfaction with all cases of auto‐segmentation performed using the pre‐built model except in only one case. In contrast, for the auto‐segmentation results obtained with the on‐site trained model, the RO1 was satisfied with approximately 80% (24/30). The RO2 expressed dissatisfaction with all cases of auto‐segmentation performed using the pre‐built model except in only one case. In contrast, for the auto‐segmentation results obtained with the on‐site trained model, the RO2 was satisfied with approximately 57% (17/30). The MP was satisfied with approximately 13% (4/30) of the auto‐segmentation cases performed using the pre‐built model. In contrast, for the auto‐segmentation results obtained with the on‐site trained model, the RO1 was satisfied with approximately 87% (26/30).

Furthermore, we summarized the average acceptance rates for each anatomical category to evaluate the model's performance in detail. Table 1 presents the average acceptance rates for each anatomical category across the three observers. For the on‐site model, the average acceptance rates were consistently high, ranging from 70.7% to 84.0% for Borders, 89.2% to 94.2% for Chambers, 66.7% to 83.3% for Great Vessels, and 65.0% to 70.8% for Coronary Arteries. In contrast, the pre‐built model showed markedly lower acceptance rates across all categories (ranging from 10.7% to 45.8%), despite its high DSC scores. The differences in acceptance rates between the two models were statistically significant across all anatomical categories (*p* < 0.05), confirming that on‐site training effectively aligns the model with clinical requirements that global geometric metrics fail to capture.

**TABLE 1:** Average acceptance rates for anatomical categories by observer and model type.

Observer	Model	Borders (%)	Chambers (%)	Great Vessels (%)	Coronary Arteries (%)
RO1	Pre‐built	20.0	36.7	45.8	39.2
	On‐site	84.0	94.2	83.3	70.8
RO2	Pre‐built	10.7	25.8	29.2	26.7
	On‐site	70.7	89.2	66.7	65.0
RO3	Pre‐built	21.3	35.8	44.2	34.2
	On‐site	77.3	92.5	75.0	68.3

In the evaluation of the pre‐built model, inter‐observer variations were not significant, whether between colleagues at the same institution (RO1 vs. MP, *p* = 0.17) or between practitioners across different institutions (RO1 vs. RO2, *p* = 0.56). In contrast, the on‐site trained model showed a significant discrepancy between RO1 and MP (*p* < 0.05), whereas the comparison between RO1 and RO2 yielded no statistically significant difference (*p* = 0.053).

The results from our in‐house developed physician‐blind test, which individually evaluated each substructure, revealed a distinct trend. For auto‐segmentation performed with the pre‐built model, the RO1 expressed satisfaction with an average of 6.4 ± 2.6 out of 17 substructures. In comparison, for the on‐site trained model, the RO1 was satisfied with an average of 14.5 ± 2.3 out of 17 substructures. For auto‐segmentation performed with the pre‐built model, the RO2 expressed satisfaction with an average of 6.0 ± 3.9 out of 17 substructures. In comparison, for the on‐site trained model, the RO2 was satisfied with an average of 13.6 ± 3.6 out of 17 substructures. The MP expressed satisfaction with an average of 8.2 ± 3.1 out of 17 substructures when using the pre‐built model, and 14.6 ± 2.3 out of 17 substructures when using the on‐site trained model. Regarding the comparison between RO1 and MP (same institution), the pre‐built model showed a significant discrepancy (*p* = 0.01), while the on‐site trained model showed no such difference (*p* = 0.91). For the cross‐institution comparison (RO1 vs. RO2), differences were nonsignificant in both the pre‐built (*p* = 0.70) and on‐site trained models (*p* = 0.22). These results mirror the overall satisfaction analysis, demonstrating that the on‐site trained model effectively aligns the evaluation criteria between observers within the same institution.

### 3.3 Correlation between geometrical similarity and physician‐blind test

#### 3.3.1 Quantified physician satisfaction

**FIGURE 2:**
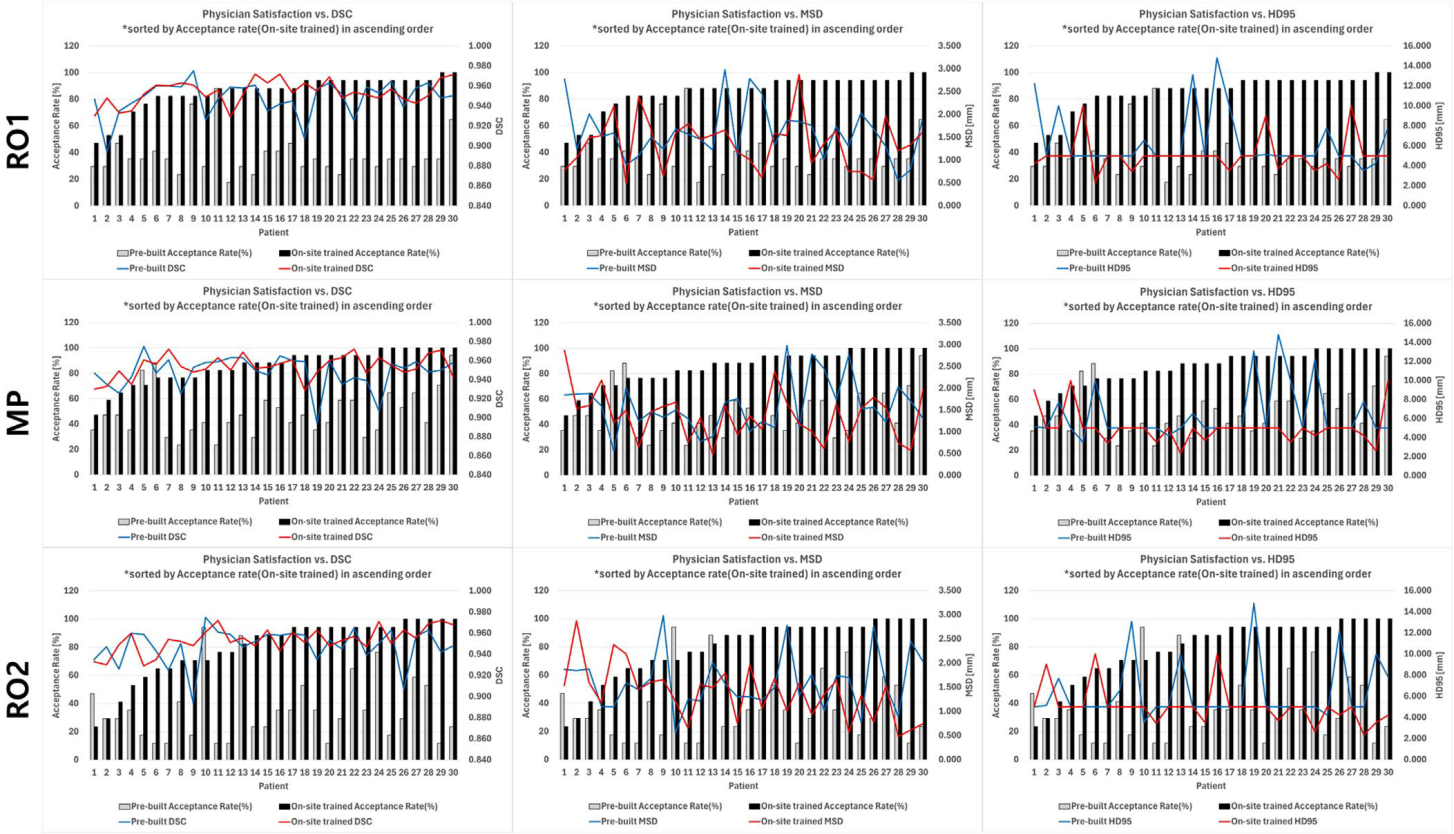
Discrepancy between physician acceptance rates and geometric similarity metrics. The bar charts show that the on‐site trained model (black) achieved a significantly higher acceptance rate across all observers (RO1, MP, RO2) compared to the pre‐built model (gray). Notably, while the acceptance rate shows an upward sorted, the corresponding geometric metrics—DSC, MSD, and HD95 (red and blue lines)—do not exhibit a proportional improvement. This suggests that the clinical acceptability of auto‐segmentation is not solely determined by conventional geometric scores, and that on‐site training enhances clinically relevant features that are not fully captured by DSC, MSD, or HD95. This visualization highlights that improved clinical acceptance through on‐site training is not necessarily accompanied by a proportional or linear increase in conventional geometric similarity scores. RO, radiation oncologist; MP, medical physicist; DSC, dice similarity coefficient; MSD, mean surface distance; HD95, 95th percentile Hausdorff Distance.

To quantitatively assess physician satisfaction, we calculated the acceptance rate across the 17 individual items of our detailed assessment. Figure 2 illustrates the relationship between this acceptance rate and the geometrical similarity indices. In all graphs, data points were arranged such that the acceptance rate for auto‐segmentation performed with the on‐site trained model increases from left to right. Interestingly, an increase in the acceptance rate of the on‐site trained model did not correspond to an increase in the acceptance rate of the pre‐built model's auto‐segmentation. Furthermore, we observed that as the acceptance rate for the on‐site trained model increased, there was no corresponding increase in the DSC for auto‐segmentation results from either the pre‐built or the on‐site trained model. Similarly, an increasing acceptance rate for the on‐site trained model did not lead to a consistent increase or decrease in the MSD or HD95 for auto‐segmentations from either the pre‐built or on‐site trained models. This trend was consistently observed for both RO and MP evaluations.

To further analyze the relationship between geometrical similarity indices and physician satisfaction, we categorized the auto‐segmentation results into two groups based on the overall satisfaction reported in the physician‐blind test: “Acceptable” and “Unacceptable.” The RO1 average acceptance rate demonstrated a significant difference of approximately 49% between the two groups (Unacceptable: 47.18 ± 17.76, Acceptable: 88.88 ± 6.90, *p*‐value: *p* < 0.001), indicating a clear distinction in satisfaction. The RO2 average acceptance rate demonstrated a significant difference of approximately 52% between the two groups (Unacceptable: 41.18 ± 23.27, Acceptable: 93.19 ± 6.87, *p*‐value: *p* < 0.001), indicating a clear distinction in satisfaction. Similarly, the MP average acceptance rate showed a significant difference of about 41% between the groups (Unacceptable: 48.88 ± 18.24, Acceptable: 87.45 ± 11.43, *p*‐value: *p* < 0.001).

While a disparity in acceptance rates existed between the ROs and the MP across the two groups, the results distinctly indicated different satisfaction levels per group for both observers. The acceptance rate showed significant differences among all observers. However, the geometrical similarity indices presented contrasting findings.

For RO1, the DSC was 0.948 ± 0.017 in the ‘Unacceptable’ group and 0.95 ± 0.011 in the ‘Acceptable’ group, with no statistically significant difference observed (*p* = 0.109). In contrast, for the MP, a significant difference was found between the ‘Unacceptable’ (0.946 ± 0.010) and ‘Acceptable’ (0.955 ± 0.010) groups (*p* = 0.019). For RO2, although the DSC values were 0.948 ± 0.016 and 0.958 ± 0.010 respectively, the difference was statistically significant (*p* = 0.005).

Regarding MSD, RO1 showed no significant difference between the “Unacceptable” (1.575 ± 0.610 mm) and “Acceptable” (1.370 ± 0.521 mm) groups (*p* = 0.179). However, significant differences were observed for both the MP (1.647 ± 0.626 mm vs. 1.332 ± 0.489 mm, *p* = 0.034) and RO2 (1.621 ± 0.564 mm vs. 1.207 ± 0.519 mm, *p* = 0.008).

**FIGURE 3:**
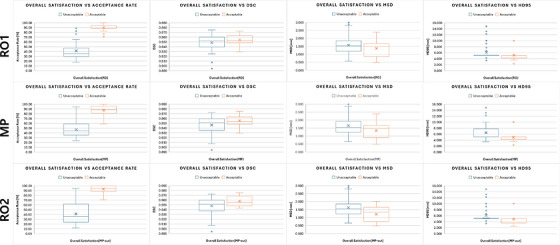
Distribution of acceptance rates and geometric metrics according to overall satisfaction. The data are divided into “Unacceptable” (blue) and “Acceptable” (orange) groups based on the overall satisfaction of each observer (RO1, MP, and RO2). While the Acceptance Rate shows a distinct difference between the two groups, the geometric metrics (DSC, MSD, and HD95) exhibit substantial overlap.

In the HD95 analysis, no significant difference was found for RO1 (6.071 ± 2.643 mm vs. 5.166 ± 1.982 mm, *p* = 0.154). Similarly, RO2 showed no significant difference (6.073 ± 2.516 mm vs. 4.877 ± 1.999 mm, *p* = 0.074), despite the numerical decrease in the “Acceptable” group. Only the MP showed a statistically significant difference in HD95 between the two groups (6.466 ± 2.852 mm vs. 4.922 ± 1.576 mm, *p* = 0.013). These results are summarized in Figure 3.

#### 3.3.2 Substructure correlation

**FIGURE 4:**
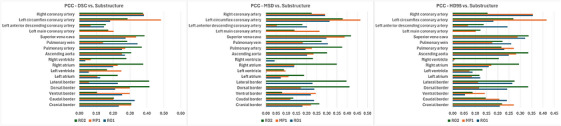
Pearson correlation coefficients between substructure acceptance rate and geometrical similarity indices. Corrected PCC values are presented for hypothesis generation only and should not be interpreted as confirmatory statistical evidence.

To evaluate which anatomical regions most significantly influence clinical judgment, PCC were calculated between the geometric metrics of 17 substructures and overall physician satisfaction (Figure 4). The analysis revealed that certain critical substructures, such as the right coronary artery, left circumflex coronary artery, and Superior vena cava, exhibited consistently higher correlations across all observers, with PCC values reaching up to 0.45. In contrast, many other regions showed negligible associations (*r* < 0.2), despite achieving high DSC scores. These findings suggest that physician acceptance is disproportionately driven by the segmentation accuracy of specific, clinically relevant landmarks rather than an aggregate geometric average.

To account for the risk of Type I errors arising from multiple comparisons, the BH correction was applied with a critical threshold of 0.002. No statistically significant correlations were identified for any observer or metric, as all calculated p‐values exceeded the BH‐adjusted threshold. The Left main coronary artery was excluded from the analysis for all observers and metrics due to a lack of variance or missing data points, resulting in undefined coefficients.

#### 3.3.3 Substructure weighting

We analyzed the relationship between the acceptability of individual substructures and overall physician satisfaction using conditional probability. Table 2 presents the calculated conditional probabilities, P (Acceptable Overall | Acceptable Substructure), indicating the likelihood that the overall result is deemed acceptable given the acceptability of each substructure. For RO1, the highest conditional probabilities were observed in the cranial border (0.86), dorsal border (0.87), and SVC (0.81), suggesting these regions act as ‘key substructure’ with a strong direct association with overall satisfaction. Similarly, for the MP, the caudal border (0.95) and AA (0.80) showed the highest levels of association. These high‐probability structures should be prioritized during the clinical QA process, as their failure is highly predictive of overall plan rejection. Notably, both observers recorded their lowest conditional probabilities in the coronary artery regions (RO1: 0.42; MP: 0.51–0.52). This indicates a more indirect association in vascular regions, where geometric success does not reliably guarantee clinical satisfaction. The expected value (*E*) is sumarized in Table 3. The expected value for RO1 was 11.31, and for the MP it was 11.65, while RO2 showed a more stringent threshold at 9.26.

**TABLE 2:** PCC analysis with Benjamini‐Hochberg (BH) correction.

Observer	Geometrical Similarity Index	Substructure	PCC	*p*‐value	BH (0.002) Correction Result
RO1	DSC	Right coronary artery	0.38	0.037	NS
	MSD	Left circumflex coronary artery	0.31	0.091	NS
	HD95	Right coronary artery	0.36	0.054	NS
MP	DSC	Left circumflex coronary artery	0.48	0.007	NS
	MSD	Left circumflex coronary artery	0.46	0.010	NS
	HD95	Left circumflex coronary artery	0.42	0.022	NS
RO2	DSC	Dorsal border	0.41	0.023	NS
	MSD	Superior vena cava	0.42	0.021	NS
	HD95	Superior vena cava	0.34	0.069	NS

Abbreviation: NS, Not Significant.

**TABLE 3:** Conditional probability of substructure acceptability for overall physician satisfaction.

		*P(B|A)*		
Category	Questionnaire	RO1	MP	RO2
Border	Cranial border	0.86	0.83	0.63
	Caudal border	0.69	0.95	0.68
	Ventral border	0.79	0.67	0.50
	Dorsal border	0.87	0.73	0.53
	Lateral border	0.77	0.70	0.57
Chamber	Left atrium	0.52	0.55	0.42
	Left ventricle	0.49	0.56	0.46
	Right atrium	0.74	0.79	0.53
	Right ventricle	0.46	0.59	0.56
Great Vessel	Ascending aorta	0.76	0.77	0.62
	Pulmonary artery	0.77	0.80	0.65
	Pulmonary vein	0.76	0.74	0.71
	Superior vena cava	0.81	0.82	0.75
Coronary artery	Left main coronary artery	0.42	0.52	0.33
	Left anterior descending coronary artery	0.75	0.61	0.44
	Left circumflex coronary artery	0.42	0.52	0.37
	Right coronary artery	0.42	0.51	0.49
Expected Value	11.31	11.65	9.26	

## 4 DISCUSSION

In this study, we developed and analyzed a novel physician‐blind test for heart auto‐segmentation to investigate the correlation between geometrical similarity indices and physician satisfaction. We performed auto‐segmentation using two models, and while their geometrical similarity was comparable, the physician‐blind test revealed divergent results in terms of physician satisfaction. Specifically, although the DSC, MSD, and HD95 showed no significant differences between the two models, the pre‐built model's auto‐segmentation yielded such low physician satisfaction that its clinical utility would be severely limited. In stark contrast, the on‐site trained model's auto‐segmentation achieved high physician satisfaction, to the extent that most results for the RO and all results for the MP were deemed immediately usable in clinical practice. The purpose of this study is not to derive a population‐level generalizable metric, but to validate a methodological framework for institution‐specific clinical decision modeling.

The central finding of this study is the marked difference in physician satisfaction despite the high and statistically indistinguishable DSC values between the pre‐built and on‐site trained models. This result quantitatively verifies the critical disconnect between high geometrical metrics and true clinical acceptability in the context of heart auto‐segmentation. Our analysis confirmed that even in cases where geometric metrics like HD95 showed no statistically significant difference between “Acceptable” and “Unacceptable” groups (e.g., *p* = 0.154 for RO1 and *p* = 0.074 for RO2), clinical judgment varied significantly. This reinforces the finding that simple geometric metrics are insufficient proxies for complex clinical judgment.

Regarding the PCC analysis for the 17 substructures, we utilized it as an exploratory tool to screen for potential correlations. The most rigorous evidence, however, is derived from the conditional probability framework, which offers a direct clinical interpretation of structural importance. The critical substructures, such as the cranial border (*p* = 0.86) and caudal border (*p* = 0.95), exhibit the highest predictive value for overall satisfaction.

Furthermore, the variation in expected values (*E*)—ranging from 9.26 for RO2 to 11.65 for the MP—highlights that clinical acceptance thresholds can differ between observers or institutions. However, the strength of this study lies in demonstrating that the same analytical process can be universally applied to establish customized, actionable guidelines. By adopting this framework, institutions can define their own minimum acceptable standards (e.g., a threshold of 11–12 successful substructures) tailored to their specific clinical stringency, thereby providing a robust QA metric for future AI model implementation.

Therefore, the clinical implications derived from the conditional probability analysis are the driving message of this paper, providing a valuable, uncorrected priority list for future model development.

### 4.1 The illusory correlation of geometrical similarity indices

The pre‐built model's low acceptance rate (RO1: 41.7%, MP: 47.0%, RO2: 41.3%) compared to its high average DSC (approximately 0.95) clearly demonstrates that global volumetric overlap metrics are inadequate indicators of clinical utility. DSC is inherently insensitive to localized, systematic errors at crucial anatomical boundaries. For instance, systematic mis‐contouring at the cranial or ventral borders constitutes only a minor volume difference relative to the entire heart volume, thus minimally impacting the global DSC value.

However, from a clinical perspective, these localized errors are highly significant. The physician's judgment is centered on the immediate usability of the contour for treatment planning. Errors at these boundaries—particularly those near the high‐dose treatment field for left breast cancer—can lead to a systematic underestimation of the MHD or high‐dose volumes (V_dose_), directly compromising safety assessments and dose‐constraint adherence.

Furthermore, our statistical analysis reinforces this disconnect. While the two‐sample *t*‐test for RO1 showed no significant difference in HD95 between “Acceptable” and “Unacceptable” groups (*p* = 0.154), RO2 also exhibited a non‐significant result (*p* = 0.074), despite a numerical decrease in the acceptable group. Even for the MP, where a statistically significant difference was observed (*p* = 0.013), the absolute numerical gap was insufficient to establish a reliable clinical threshold. This lack of consistent, robust significance across observers for both DSC and HD95 emphasizes that simple geometric fidelity is an insufficient proxy for the comprehensive clinical assessment required in auto‐segmentation.

Specifically, the discrepancy observed at the cranial and caudal borders‐where high DSC scores often mask significant longitudinal errors‐is likely attributable to the limitations of the 2D slice‐based FCDN architecture used in this study. Anatomically, the superior and inferior limits of the heart involve rapid changes in cross‐sectional geometry, which are inherently more difficult for a 2D‐based model to predict with high precision compared to the more stable mid‐ventricular chambers. Furthermore, due to the mathematical bias of DSC, which is heavily weighted toward larger volumes, these clinically critical errors in small‐volume border regions have a negligible impact on the overall score.^21^ This confirms that a high global DSC can be “illusory,” as it fails to account for the localized, 2D‐driven inaccuracies that dictate a physician's decision to accept or reject a contour.^22^

### 4.2 Clinical importance of substructures: Conditional probability analysis

Based on this conditional probability framework, the calculated Expected Value (*E*) of acceptable substructures required for overall satisfaction was 11.31 for RO1 and 11.65 for the MP. These values suggest a minimum clinical threshold of approximately 12 out of 17 substructures to achieve an “Acceptable” rating. Notably, RO2 exhibited a more stringent threshold with an E value of 9.26. While these absolute values vary between observers and institutions, they demonstrate that our methodology provides a reproducible process for establishing customized, actionable standards tailored to specific clinical requirements.

Our analysis identified several critical structures with high conditional probabilities (P(B|A) > 0.80), such as the Cranial border (0.86), Dorsal border (0.87), and SVC (0.81) for RO1, and the Caudal border (0.95) and Ascending Aorta (0.80) for the MP. These structures represent the baseline anatomical fidelity required for clinical acceptance. Mis‐contouring these areas, which define the heart's position and its relationship with major vessels, is highly predictive of an overall ‘Unacceptable’ verdict. Prioritizing these high‐probability structures during manual QA ensures the prevention of systematic dose underestimation, a critical factor in reducing long‐term cardiac toxicity.

Furthermore, the lowest conditional probability values were consistently found in the coronary artery regions, with values as low as 0.42 for RO1 (LCA, LCx, RCA) and 0.51–0.52 for the MP. This lower probability indicates a more indirect or complex association; in these small, intricate structures, achieving geometric success (e.g., high DSC) does not reliably guarantee clinical satisfaction. This finding is particularly relevant in left breast cancer radiotherapy, where the dose to coronary arteries is strongly correlated with cardiac toxicity. The sensitivity of these regions to even minor clinical discrepancies disproportionately impact final judgment, further validating the failure of global metrics like DSC to capture clinical risk.

**FIGURE 5:**
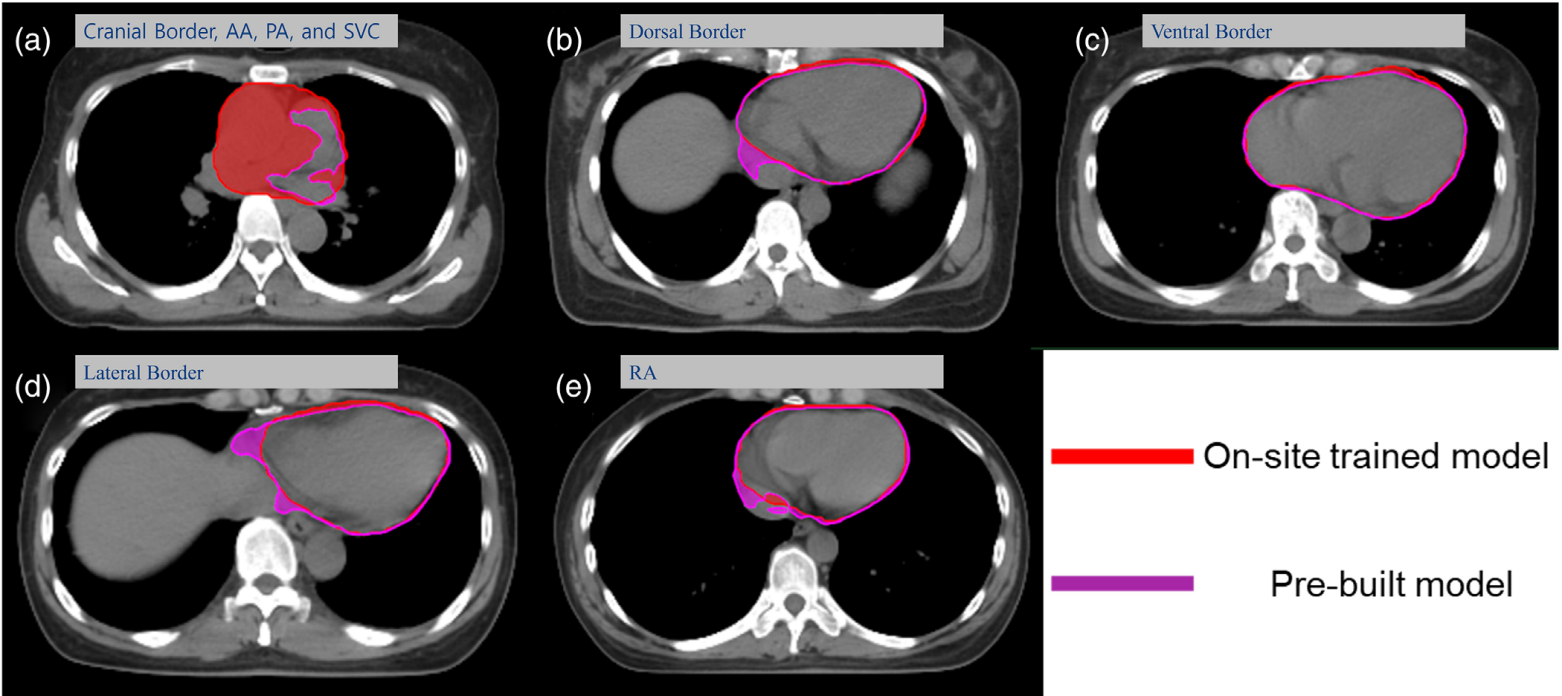
Examples of heart auto‐segmentations highlighting key substructures for clinical satisfaction. This figure illustrates substructures identified as critical through conditional probability analysis. (a) Cranial Border (RO1:0.86, MP: 0.83, RO2: 0.63), AA (RO1: 0.76, MP: 0.77, RO2: 0.62), PA (RO1:0.77, MP: 0.80, RO2: 0.65), and SVC (RO1:0.81, MP: 0.82, RO2: 0.75). (b) Dorsal Border (RO1:0.87, MP: 0.73, RO2: 0.53). (c) Ventral Border (RO1:0.79, MP: 0.67, RO2: 0.50). (d) Lateral Border (RO1:0.77, MP: 0.70, RO2: 0.57). (e) RA (RO1:0.74, MP: 0.79, RO2: 0.53). Abbreviations: RO, radiation oncologist; MP, medical physicist; AA, ascending aorta; PA, pulmonary artery; SVC, superior vena cava; RA, right atrium.

As shown in Figure 5, there are distinct anatomical variations in the critical structures that influence clinical satisfaction. Several key substructures with high conditional probabilities (*P* > 0.80), such as the Cranial border, AA, and SVC, are in the superior region of the heart (Figure 5a). Overall, the pre‐built model showed significant discrepancies compared to the on‐site trained model, particularly in the superior and right regions of the heart. These differences likely stem from the specific characteristics of the training datasets. Consequently, our findings suggest that for successful auto‐segmentation, auto‐segmentation tools should be refined or trained using institution‐specific patient data to align with local clinical standards.

Moreover, as illustrated in Figures 5b and e, our analysis revealed subtle but important differences in professional priorities. For instance, RO1 placed higher importance on the dorsal border (*P* = 0.87), whereas the MP demonstrated a stronger requirement for the complete delineation of the right atrium (*P* = 0.79) and especially the caudal border (*P* = 0.95). These professional preferences are reflected in the expected values (*E*), which were 11.31 for RO1 and 11.65 for MP, suggesting that both observers require approximately 12 out of 17 substructures to be acceptable for overall satisfaction.

Notably, RO2 exhibited a lower expected value of 9.26, indicating a more stringent internal threshold for clinical acceptance. These variations underscore that professional judgment is influenced by distinct clinical experiences and institutional training. However, the consistent application of our conditional probability framework across all observers demonstrates that this methodology can be used as a standardized tool to derive customized clinical guidelines, allowing each institution to define its own minimum acceptable criteria based on its specific professional requirements.

### 4.3 Limitations of this study

This study has several limitations that should be acknowledged. First, the evaluation of clinical satisfaction was conducted by a relatively small number of observers (two radiation oncologists and one medical physicist). Although these observers were selected from different institutions to ensure objectivity, their perspectives may not fully represent the diverse clinical judgment criteria of the broader radiation oncology community. Second, our assessment of clinical acceptability was primarily binary (Yes/No), which may not capture the subtle nuances of “acceptability with minor revisions” often encountered in real‐world clinical workflows. Future studies incorporating a more granular Likert scale could provide further insights. Third, since the auto‐segmentation models used were proprietary algorithms (FCDN) within commercial software, the specific deep‐learning architectures and training parameters were not fully controllable, limiting our ability to perform a detailed technical analysis of why certain errors occurred. Finally, while we emphasize the “illusory correlation” of the DSC, it remains a vital metric for assessing global volumetric similarity. Our findings should be interpreted not as a rejection of DSC, but as a call for a more comprehensive QA framework that integrates conditional probability and anatomical prioritization.

## 5 CONCLUSION

This study elucidates a significant quantitative disconnect between conventional geometric similarity indices—such as DSC, MSD, and HD95—and actual clinical acceptability in heart auto‐segmentation. This is what often leads to “illusory correlations.” Our findings reveal that high volumetric overlap metrics are insufficient predictors of clinical utility, as physician satisfaction is driven by the precise delineation of specific critical substructures rather than aggregate geometric averages.

The analytical framework developed herein, based on conditional probability and expected values, serves as a robust QA tool for the systematic evaluation of AI‐generated contours.

First, the framework functions as a clinical prioritization engine. By statistically identifying critical substructures (e.g., the cranial/caudal borders and SVC) that disproportionately influence overall satisfaction, the tool allows clinicians to focus their limited review time on high‐impact regions. Furthermore, it identifies high‐sensitivity areas, such as the coronary arteries, where geometric metrics frequently fail to reflect clinical risk, thereby alerting medical staff to the necessity of manual verification regardless of favorable similarity scores.

Second, this methodology acts as a quantitative validation gateway for model deployment. By establishing an institutional “Expected Value” (e.g., a threshold of 11–12 acceptable substructures), the framework provides an objective benchmark to determine the “Clinical Readiness” of an auto‐segmentation model. This ensures that only models meeting a verified standard of clinical professional judgment are integrated into the active treatment workflow.

In conclusion, this research advocates for a paradigm shift in AI evaluation from simple geometric fidelity to probability‐based clinical risk management. The proposed QA tool provides a reproducible, evidence‐based methodology for institutions to define and uphold their own standards of clinical excellence in the era of AI‐driven radiotherapy.

## AUTHOR CONTRIBUTIONS

**Jae Choon Lee**: Conceptualization; Methodology; Formal analysis; Investigation; Writing—Original Draft. Visualization; Project administration. **Eun Jeong Heo**: Investigation; Data Curation. **Song Heui Cho**: Resources. **Dong yun Lee**: Software. **Kyung Hwan Chang**: Writing—Review & Editing. **Jang Bo Shim**: Writing—Review & Editing. **Nam Kwon Lee**: Resources. **Suk Lee**: Writing—Review & Editing; Visualization; Supervision; Project administration; Funding acquisition.

## CONFLICT OF INTEREST STATEMENT

The authors have no relevant conflicts of interest to disclose.
